# Genomic Sequence Resource of *Talaromyces albobiverticillius*, the Causative Pathogen of Pomegranate Pulp Rot Disease

**DOI:** 10.3390/jof9090909

**Published:** 2023-09-07

**Authors:** Tan Wang, Shuchang Chen, Qiuhong Niu, Guangling Xu, Chenxu Lu, Jin Zhang

**Affiliations:** 1 School of Life Science and Agricultural Engineering, Nanyang Normal University, Nanyang 473061, China; 2 School of Forestry and Biotechnology, Zhejiang A&F University, Hangzhou 311300, China

**Keywords:** *Talaromyces*, whole genome sequencing, pigment production

## Abstract

*Talaromyces albobiverticillius*, a prominent pathogen responsible for pomegranate pulp rot disease, inflicts significant damage on *Punica granatum* L. Besides its pathogenicity, this fungus possesses the potential to produce substantial amounts of red pigments, making it promising for industrial applications. This study presents the genome annotation of *T. albobiverticillius* field strain Tp-2, isolated from pomegranates. The genome assembly, generated through a combination of Oxford Nanopore and Illumina sequencing reads, yielded a high-quality assembly with 14 contigs, featuring an N50 length of 4,594,200 bp. The complete genome of strain Tp-2 spans 38,354,882 bp, with a GC content of 45.78%. Importantly, the assembly exhibits remarkable integrity, with 98.3% of complete Benchmarking Universal Single-Copy Orthologs validating genome completeness. Genome prediction analysis reveals the presence of 10,380 protein-coding genes. To our knowledge, this study is the first report on the genome sequence of *T. albobiverticillius*, offering valuable insights into its genetic variation and molecular mechanisms of pigment production.

## 1. Introduction

The genus *Talaromyces* was first described by Benjamin in 1955 as a sexual state of *Penicillium* [1]. In 2011, Professors Houbraken and Samson redefined the genus *Talaromyces* based on genetic identification and the priority principle of international nomenclature regulations [2]. The number of *Talaromyces* species is rapidly increasing, owing to the availability of reliable and complete sequence data contributed from around the world [3]. *Talaromyces* includes medically important species, such as *Talaromyces marneffei*, which are associated with acquired immunodeficiency syndrome caused by human immunodeficiency virus infections [4]. Moreover, *Talaromyces* comprises species known for their production of various bioactive compounds, including anticancer agents [5], antibacterial and antifungal compounds [6], antioxidative compounds [7], enzymes [8,9], and natural colorants [10].

Among these pigment-producing species, *Talaromyces albobiverticillius* generates a substantial number of nontoxic Monascus-like azaphilone red pigments [11,12,13] which can be used as natural colorants in industrial applications. Meanwhile, pomegranate fruit pulp rot disease caused by *T*. *albobiverticillius* has been found in Italy [14] and China [15], resulting in significant production losses. The cultivation of pomegranate (*Punica granatum* L.) has recently sparked growing interest due to its potential impact on human health. Losses experienced both during cultivation and after harvesting are the main concerns in this valuable production chain. The main postharvest diseases of pomegranates were caused by latent pathogens that infect the fruits during the blooming phase (*Coniella granati*, *Alternaria* spp., *Botrytis* spp.), and secondarily by wound pathogens (*Penicillium* spp., *Talaromyces* spp., and *Aspergillus* spp.) that affect the fruits during the processing from harvest until the conclusion of storage [16,17]. In 2020, instances of postharvest disease in pomegranates attributed to *Talaromyces* spp. were documented in Serbia [18]. Hence, within the pomegranate sector, investigating the genomic genetic data and the pathogenic mechanisms of *Talaromyces* spp. has become highly imperative.

However, no information on the *T*. *albobiverticillius* genome is currently available. Herein, we present the first genome assembly for *T*. *albobiverticillius* and analyze its genomic features using Oxford Nanopore and Illumina sequencing technologies. The availability of this genomic data is likely to aid research on evolution, pigment manufacturing systems, infection mechanisms, and disease management.

## 2. Materials and Methods

### 2.1. Fungal Material and Culture Conditions

The strain Tp-2 used in this study was originally isolated from contaminated pomegranate fruits gathered from Xichuan, a city in China’s Henan province. The surface of the colony was grayish-green with a claret-red exudate. Fuchsia pigments are normally secreted into potato dextrose agar (PDA) medium. *T*. *albobiverticillius* was identified as the strain Tp-2 based on morphological traits and a multigene phylogenetic analysis [15]. Before DNA extraction, the strain was grown on PDA medium for 5 days at 25 °C. Mycelium strain Tp-2 was harvested and immediately frozen in liquid nitrogen for genomic DNA extraction.

### 2.2. DNA Extraction and Genome Sequencing

The genomic DNA of the Tp-2 strain was extracted from 5-day-old fresh mycelia using the sodium dodecyl sulfate method [19]. Briefly, 1 g of fresh mycelia was collected and combined with 900 μL of 10% SDS lysis buffer. To this mixture, 100 μg proteinase K per ml of emulsion solution was added, and the solution was incubated for 1 h at 50 °C. Subsequently, 350 μL of neutral saturated salt solution (NaCl) per ml was introduced into the tube. The tube was then centrifuged at 590× *g* for 15 min, resulting in DNA remaining exclusively in the aqueous phase. The resultant aqueous phase was mixed with two volumes of absolute ethyl alcohol. After removing the supernatant, the DNA pellet was washed once with 75% ethanol and the DNA was precipitated out of the solution by centrifugation at 9500× *g* for 5 min. The isolated DNA was detected using 1% agarose gel electrophoresis and quantified using the Qubit 2.0 Fluorometer (Thermo Scientific, Waltham, MA, USA). Libraries for nanopore sequencing were constructed with a 10-kb insert size of genomic DNA. First, large DNA fragments were recovered using the Blue Pippin automatic nucleic acid fragment recovery system and then repaired. Barcodes were added using the PCR-free method of the EXP-NBD104 kit (Oxford Nanopore Technologies, Oxford, UK). The size of the fragments was subsequently determined using the AATI automatic mixer (Agilent Technologies, Santa Clara, CA, USA). Finally, the SQK-LSK109 connection kit (Oxford Nanopore Technologies, Oxford, UK) was used to link the adapter, completing the construction of the 10-K library. Libraries for Illumina sequencing were generated using the NEBNext^®^ UltraTM DNA Library Prep Kit for Illumina (New England Biolabs, Ipswich, MA, USA) following the manufacturer’s recommendations, with index codes added to attribute sequences to each sample. Briefly, DNA samples were sonicated to a size of 350 bp, and then DNA fragments were end-polished, A-tailed, and ligated with full-length adapters for Illumina sequencing, followed by further PCR amplification. The PCR products were purified using AMPure XP Bead-based reagent (Beckman, Indianapolis, IN, USA), and the libraries were analyzed for size distribution using an Agilent 2100 Bioanalyzer (Agilent Technologies, Santa Clara, CA, USA) and quantified using real-time PCR. The whole genome of the Tp-2 strain was sequenced using the Nanopore PromethION 48 platform and Illumina NovaSeq PE150 (Novogene Bioinformatics Technology, Beijing, China).

### 2.3. Genome Assembly and Prediction

The nanopore data was assembled using the Flye software (version 2.9-b1768), which produced preliminary assembly results. The splicing results were corrected using Racon (version 1.4.13) and Pilon (version 1.22) software, which produced final assembly results. The Benchmarking Universal Single-Copy Orthologs (BUSCO version 3.0.2) assessment used a single-copy orthologous gene library, and software such as Metaeuk and Hmmer were used to evaluate the assembled genome to assess the assembled genome’s integrity. The Augustus (version 2.7) program was used to retrieve predicted coding genes.

### 2.4. Phylogenetic Analysis

The NCBI Genome Database was used to retrieve the annotated gene sequences from additional species of phylogenetically related *Talaromyces* taxa. Genome alignments were performed in MUMmer 3 (version 3.2.2) using an all-against-all comparison with the default parameters. Then, the tool OrthoFinder (version 2.4.0) was used to identify key orthologs [20]. With the help of IQ-Tree (version 2.2.0), a phylogenetic tree was constructed [21].

### 2.5. Dispersed Repeat Sequences and Noncoding tRNA Annotation

Dispersed repeat sequences were masked throughout the genome using the Repeat Masking option (RepeatMasker v.4.0.5) [22], which was implemented in OmicsBox software v.1.4.12. Tandem repeats were analyzed using TRF (version 4.07b) [23]. Transfer RNA (tRNA) genes were predicted using tRNAscan-SE (version 1.3.1) [24], while ribosomal RNA (rRNA) genes were analyzed using rRNAmmer (version 1.2) [25]. For small RNA (sRNA) analysis, we initially aligned and annotated the data with the Rfam database [26]. Subsequently, the CMsearch program (Version 1.1rc4) was employed to determine the final sRNA annotations.

### 2.6. Gene Annotation and Functional Analyses

Basic annotations of predicted genes were performed using seven major databases, including Gene Ontology (GO), Kyoto Encyclopedia of Genes and Genomes (KEGG), Clusters of Orthologous Groups (KOG), Non-Redundant Protein Database (NR), Transporter Classification Database (TCDB), Pfam, and Swiss-Prot [27,28,29,30,31]. We comprehensively predicted whether the protein sequence was a secreted protein using SignalP (Version 4.1) [32] and TMHMM (Version 2.0c) algorithms to determine whether the genome sequences included signal peptides and transmembrane structures. Meanwhile, we analyzed the secondary metabolism gene clusters using antiSMASH (version 5.1) [33] and causative genes using Pathogen–Host Interactions (PHI, version 4.12) and Database of Fungal Virulence Factors (DFVF) [34]. The Carbohydrate-Active Enzymes (CAZymes) Database [35] predicted CAZymes.

## 3. Results

### 3.1. Genome Assembly and Genomic Characteristics

**Figure 1:**
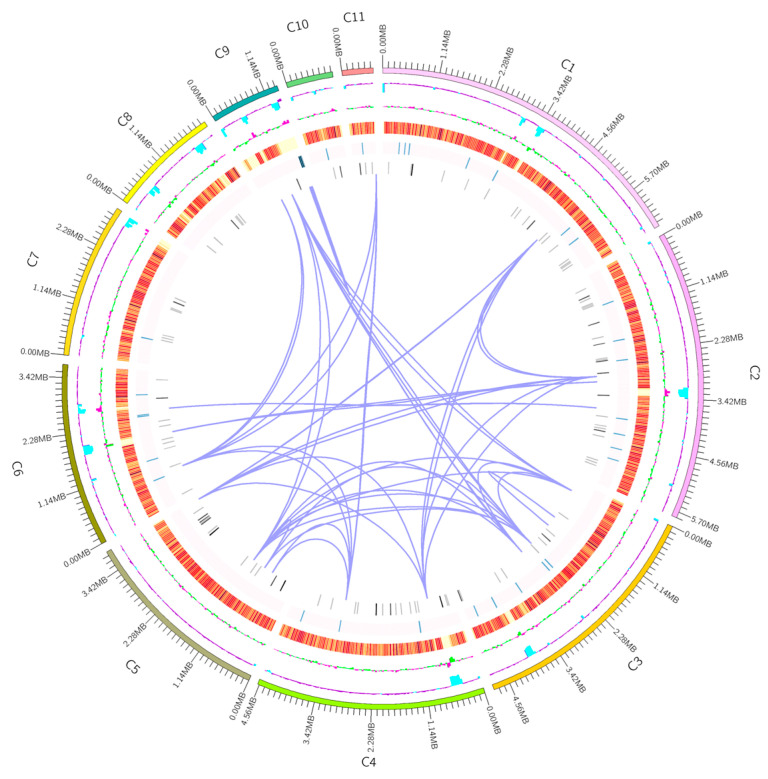
Genomic landscape of *Talaromyces albobiverticillius* strain Tp-2 visualized using Circos software (version 0.69-9). The outermost circle represents the location coordinates of the genome sequence. Moving inward, the subsequent circles depict GC content, GC-skew, density of coding genes, rRNA, snRNA, and tRNA. The curves connecting the different regions in the central circle represent gene duplications.

We present the first de novo genome sequencing of the Tp-2 strain of *T*. *albobiverticillius* using short- and long-read technologies. In this study, nanopore sequencing produced 6,588,890,037 bp of raw read data and Illumina paired-end sequencing produced 4844 Mb of raw read data (described in Section 2.2). The assembled Tp-2 genome had a length of 38,354,882 bp and a GC content of 45.78%. The BUSCO analysis (described in Section 2.3) showed that 98.3% of the BUSCOs were complete and that 98.3%, 0%, and 0.3% of the BUSCOs were single-copy, duplicated, and fragmented, respectively (Figure S1). More characteristics of the assembled genome are presented in Table 1 and Figure 1.

**Figure 2:**
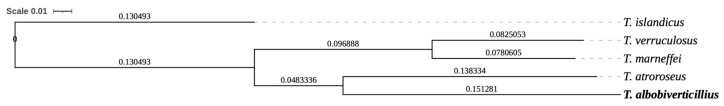
A phylogenetic tree of five *Talaromyces* species constructed based on whole genome sequence data using IQ-Tree.

The assembly statistics of *T*. *albobiverticillius* and its phylogenetically related species are presented in Table 2. To establish the evolutionary relationship among the *Talaromyces* species, pairwise comparisons were conducted using the genomes of *T*. *albobiverticillius*, Tp-2, *T*. *atroroseus* IBT 11181, *T*. *islandicus* WF-38-12, *T*. *marneffei* ATCC 18224, and *T*. *verruculosus* TS63-9 (described in Section 2.4). These assessments revealed 3777 single-copy orthologous genes shared among these five species. Additionally, the genome data of *T*. *atroroseus*, *T*. *islandicus*, *T*. *marneffei*, and *T*. *verruculosus* contained 6396, 5809, 5871, and 6493 one-to-one orthologous genes, respectively. The phylogenetic analysis illustrated that *T*. *atroroseus* exhibited a closer evolutionary relationship to *T*. *albobiverticillius* than to other species (Figure 2).

### 3.2. Gene Prediction and Functional Annotation

We employed the Augustus 2.7 program to predict fungal genes and obtained 10,380 genes (Table 3). Subsequently, through rRNAmmer and tRNAscan-SE analyses, we predicted 204 noncoding RNAs, which included 47 ribosomal RNAs (rRNA) and 157 transfer RNAs (tRNA) (described in Section 2.5). Our investigation led to the identification of 7412 proteins harboring Pfam domains, 8844 genes participating in various KEGG pathways, and 2160 KOG genes (described in Section 2.6). Additionally, antiSMASH analysis revealed 214 Type I polyketide synthases (T1PKSs) genes closely associated with red pigment synthesis in *T*. *albobiverticillius*. Furthermore, we mapped 750 genes to the CAZyme database and encoded CAZymes. A total of 227 genes were assigned to the cytochrome P450 family. Notably, 400 virulence genes associated with pathogenesis were predicted via DFVF. Additionally, our search of the PHI database revealed 1332 associated genes. Moreover, we identified 2057 transmembrane proteins, 638 transport proteins, and 671 secreted proteins using the TMHMM, TCDB, and SignalP databases, respectively. These genes are involved in the pathogenesis of fungi and may play an important role in the pathogenesis of *T*. *albobiverticillius*.

#### 3.2.1. GO Annotation

The database categorizes genes into three groups based on their roles, including cellular component, molecular function, and biological process (described in Section 2.6). Gene function classification analysis of *T*. *albobiverticillius* revealed that most genes participating in biological processes were involved in metabolic (3988), cellular (2971), and single-organism (2657) activities, as well as localization (1491) processes (Table S1). Cell (2806), membrane (2589), organelle (1444) portions, macromolecular complex (646), and membrane-enclosed lumen (139) were the most common gene categories listed in the cellular component category. Genes were mostly involved in catalytic activity (4117), binding (3660), transporter activity (785), structural molecule activity (128), and signal transducer activity (54).

#### 3.2.2. Annotation of CAZymes

The CAZymes database is a professional database related to carbohydrate enzymes, including those capable of catalyzing carbohydrate degradation and modification as well as related enzyme families for biosynthesis (described in Section 2.6). A total of 750 genes encoding putative CAZymes were identified in *T*. *albobiverticillius* (Table S2). Under the five categories and one structural domain, multiple families have been established. Based on the results, GHs were the most prevalent family, with 105 enzymes (427 genes). The next most abundant category was GTs, with 37 enzymes (121 genes), followed by the AA family with 14 enzymes (60 genes). The other three categories, namely CBMs, CEs, and PLs, were relatively less numerous, containing 12 (91 genes), 10 (40 genes), and 8 (11 genes) enzymes, respectively (Table 4). The main GH subfamilies detected were chitinases (GH18), *β*-glucosidases (GH3), polygalacturonases (GH28), invertase (GH32), *α*-L-rhamnosidase (GH78), *α*-1,6-mannanase (GH76), *α*-glucosidase (GH31), α-galactosidase (GH27), and α-1,2-mannosyltransferase (GH22), which are listed in descending order of the quantity of genes. Within the GT families, the most abundant were those involved in N-acetyllactosaminide (GT31), UDP-glucuronosyltransferases (GT1), α-1,2-mannosyltransferase (GT22), and α-trehalose-phosphate synthase (GT20). Among the AA family, the most predominant subfamilies were oxidoreductases (AA1) and glucooligosaccharide oxidases (AA7). Regarding the CE subfamilies, acetylesterase (CE16) and acetyl xylan esterase (CE5) were the most abundant, while the chitopentaose-binding (CBM50) family was the most prominent in the CBM category.

#### 3.2.3. Annotation of Transport Proteins

The Transporter Classification Database (TCDB) is a classification system for predicting membrane transport proteins (described in Section 2.6). In *T*. *albobiverticillius*, 638 genes encoding transport proteins were discovered, with 18 genes (2.8%) sharing more than 80% similarity (Table S3). The electrochemical potential-driven transporters (263 genes) were the most prominent group, followed by the main active transporters (163 genes) and channels and pores (73 genes).

#### 3.2.4. Annotation of Secreted Proteins

Secreted proteins are those that are produced within a cell and are subsequently released to exert their effects outside of it. The N-terminus of secreted proteins typically harbors a signal peptide, consisting of approximately 15 to 30 amino acids. The SignalP prediction tool was utilized to annotate whether a protein sequence includes a signal peptide structure. Furthermore, the TMHMM tool was employed to determine the presence of transmembrane structures within a protein sequence. Ultimately, proteins that possess signal peptide structures while lacking transmembrane structures are singled out as secreted proteins. Following an analysis utilizing the SignalP 4.1 program (described in Section 2.6.), we have identified a total of 671 secreted proteins within the entirety of the genomic dataset (Table S4). Among these, the count of proteins with a prediction score surpassing 0.8 amounted to 190, constituting 28.3% of all the predicted secreted proteins. This observation signifies a higher likelihood of these proteins containing signal peptides.

#### 3.2.5. Annotation of Putative Virulence-Associated Genes

The DFVF is a comprehensive database of fungal virulence factors, containing 2058 known pathogenic genes. Each gene is associated with details about the disease it causes and the host it targets, along with Pfam functional domain annotations and GO annotation information. By comparing the results obtained from the Diamond program (described in Section 2.6), we identified 400 homologous virulence genes, of which 38 genes (9.5%) shared more than 80% identity (Table S5). These highly homologous genes include polyubiquitin (CAA76783) in *Candida albicans* (100%), histone 3 (ACZ56011) in *Fusarium* sp. (97.7%), MAP kinase (AAS20192) in *Alternaria brassicicola* (95.1%), tubulin alpha-1 subunit (EAL87967) in *Aspergillus fumigatus* (95.1%), Ras-related protein (EAQ71072) in *Pyricularia oryzae* (86.7), argininosuccinate lyase (BAB40769) in *Fusarium oxysporum* (86.3%), Rab/GTPase (CAC41973) in *Colletotrichum lindemuthianum* (83.6%), and catalase (AAN04057) in *Talaromyces marneffei* (83.6%). These genes play critical roles in the pathogenicity of their respective species.

The Pathogen–Host Interactions (PHI) database contains verified information on virulence-associated genes that regulate pathogen host interactions. Based on the PHI analysis (described in Section 2.6), we discovered 1332 PHI putative genes in the whole genome of *T*. *albobiverticillius*, 462 of which do not affect pathogenicity (Table S6). Furthermore, 550 genes were linked to decreased virulence and 100 gene deletions entirely failed to produce illness. There were 23 increased virulence (hypervirulence) genes and 75 fatal genes, all of which were required for the pathogen’s growth, metabolism, and reproduction. Only 15 and 3 of the 1332 putative genes were effectors and chemical targets, respectively. *Fusarium graminearum* and *Magnaporthe oryzae* had the highest number of homologous genes related to virulence (118 and 117 genes, respectively), followed by *Aspergillus fumigatus* with 83 genes (Table 5).

#### 3.2.6. Annotation of Secondary Metabolites Gene Clusters

**Figure 3:**
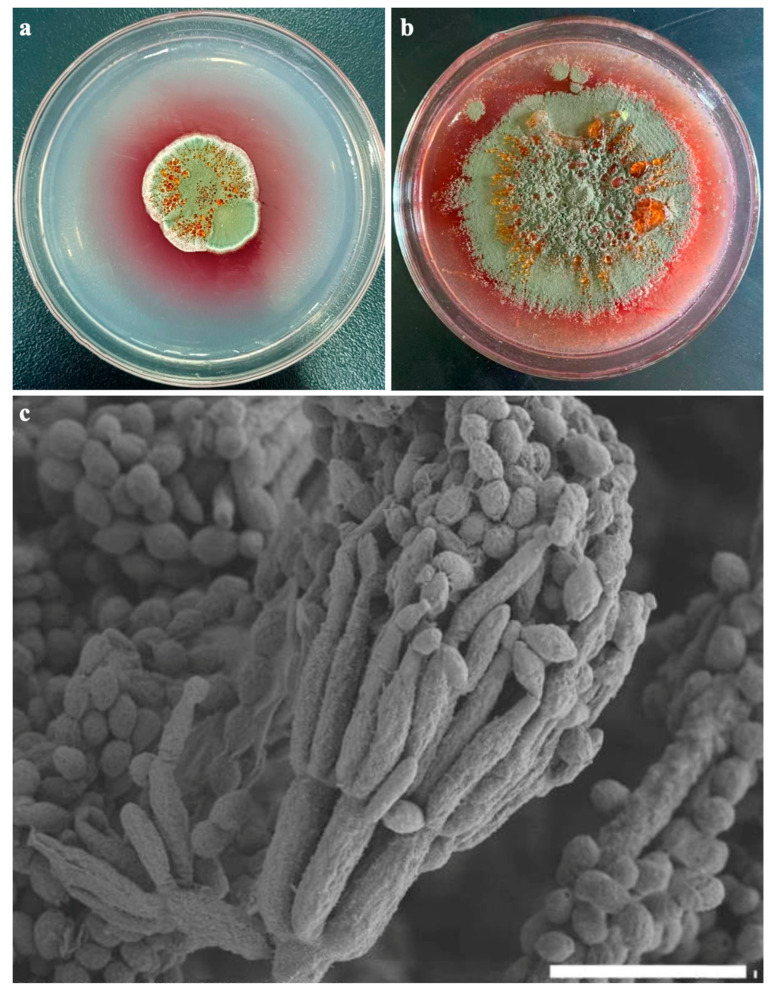
Colonies and conidia morphology of the Tp-2 strain under an electron microscope. (**a**) A 5-day-old colony of the Tp-2 strain cultivated on a PDA plate (90 mm in diameter) at 28 °C. (**b**) A 14-day-old colony, revealing the secretion of claret-red pigments into the medium. (**c**) Conidia and strigmata visualized under a scanning electron microscope. Scale bar = 10 μm.

Most secondary metabolites have complicated molecular structures, such as polyketides and nonribosomal peptides, which are produced by the enzymes polyketide synthase (PKS) and nonribosomal peptide synthase (NRPS), respectively. One of the crucial secondary metabolites produced by fungi is pigment, which is often produced following active cell development. Fungal pigments have reportedly been shown to have bioactive properties, which is significant for screening new drugs [13]. It can be seen from Figure 3 that the 5-day-old colony (Figure 3a) of the Tp-2 strain was cultured on a PDA plate (90 mm in diameter). As the culture time increased, the 14-day-old colony (Figure 3b) revealed the secretion of claret-red pigments into the medium. According to previous research, *T*. *albobiverticillius* was known to produce pigments that resembled azaphilones (Figure 3). FK17-P2b1, monascusone A, PP-Y, monascorubrin, PP-O, talaralbols A, and talaralbols are the names of the seven azaphilone pigments that have been isolated and reported [36]. The biosynthesis of azaphilone involves both the fatty acid synthesis route and the polyketide pathway.

There were 62 gene clusters (724 genes) involved in the secondary metabolism of *T*. *albobiverticillius* (Table S7). The gene clusters identified on the *T*. *albobiverticillius* genome encode various types of secondary metabolite biosynthetic pathways, including 18 T1PKS (type 1 PKS), 15 NRPS-like, 9 NRPS, 9 terpenes, 4 NRPS-T1PKS, 4 NRPS-like-T1PKS, 1 *β*-lactone, 1 NRPS-*β*-lactone, and 1 other (described in Section 2.6). Using the antiSMASH program, we found six PKS genes with 100% similarity in the T1PKS gene cluster. These PKS genes encode the secondary metabolites YWA1 from *Aspergillus oryzae*, alternariol from *Parastagonospora nodorum*, cichorine from *Aspergillus nidulans*, ACT toxin II from *Alternaria alternata*, and monascorubrin from *Talaromyces marneffei*. Upon analyzing these sequences, we discovered that the genome of *T*. *albobiverticillius* contained homologous genes that encode various secondary metabolites, such as toxins and pigments.

## 4. Discussion

*Talaromyces* now comprises 171 species, a notable increase since the genus’ reclassification in 2011, demonstrating the genus’ great diversity and widespread interest. In total, 21 species of *Talaromyces* have had their whole genomes sequenced as of the end of August 2023, according to the NCBI Genome Database. In this study, the genomic information of *T*. *albobiverticillius* was compared with that of four additional significant species of the *Talaromyces* genus. It was discovered that GC content (46%), the number of predicted genes (9523–11,447), and the number of homologous genes were virtually the same, suggesting that these markers are highly conserved throughout species. The significant variation in the genome assembly size (28.6–38.4 Mb) could be due to various sequencing techniques, depths, and assembly techniques. The comparison results of the genome phylogenetic tree revealed that *T*. *albobiverticillius* and *T*. *atroroseus* belonged to the same branch and that *T*. *marneffei* and *T*. *verruculosus* belonged to the same branch.

Remarkably, *T*. *atroroseus*, which happens to be the nearest relative to *T*. *albobiverticillius*, is renowned for its ability to produce monascus pigments, which are highly applicable in the food industry [37]. A draft genome of a prospective fungal cell factory, *T*. *atroroseus* IBT 11181 (CBS 123796), was reported in 2017 [38]. Likewise, *T*. *verruculosus* has garnered recognition for its exceptional ability to produce cellulases and antibacterial compounds [39]. In 2016, researchers unveiled the preliminary genome of *T*. *verruculosus* strain TS63-9, known for its remarkably high pectinase activity. *T*. *marneffei* is native to Southeast Asia and South China and serves as an opportunistic infectious agent in individuals with weakened immune systems [40]. The prevalence of systemic *T*. *marneffei* infection has experienced a significant surge in recent times, aligning with the rising occurrence of HIV infections [41]. *T*. *islandicus* is a globally distributed storage mold that is associated with the deterioration of grain crops following harvest, posting a threat to their quality [42]. In 2015, researchers successfully conducted whole genome sequencing and generated a preliminary version of *T*. *islandicus*, focusing on the WF-38-12 strain [43]. This particular strain exhibits a remarkably adaptable metabolism, which is evident through its ability to produce a wide variety of biopolymer-degrading enzymes, mycotoxins, and anthraquinones. These traits collectively present a diverse array of potential applications in various industrial fields. Interestingly, *T*. *islandicus* contains the most unassigned genes (515) out of all analyzed species, which is consistent with the finding that *T*. *islandicus* was farthest from the others in the phylogenetic tree. The evolutionary tree’s homology relationship might be explained by the host species or by the strain specificity within a given host species.

To predict genes, we employed various databases, including GO, KEGG, KOG, and NR. We also attempted to accurately and comprehensively annotate the gene functions of *T*. *albobiverticillius*. It was discovered that the NR database, with a total of 9782 genes, contained the most annotated genes. These findings exhibit resemblances to earlier genome-wide analyses conducted on closely related species.

*T*. *albobiverticillius* was a long-lasting latent pathogen on pomegranates, according to earlier research. On pomegranates, conidia infect epidermal cells in the stigma, hair, and silks. Along with the parenchyma and vascular cells in the silks, hyphae spread infection toward the ovary. The outcome demonstrated that *T*. *albobiverticillius* was capable of directly infecting silks and causing pulp rot through the silk channel. The fruits had no visible symptoms, although some of their pulp had turned brown before harvest. Fruits collected from afflicted orchards developed brown lesions on their peels after long-term (30–40 days) storage under ambient conditions, from which *T*. *albobiverticillius* could be isolated. To date, there has been no investigation into the pathogenic genes of *T*. *albobiverticillius*. Accordingly, the genome’s pathogenicity-related genes, such as polyubiquitin, MAP kinase, catalase, and Ras protein, were also discovered by comparing the genomes to the those in the DFVF and PHI databases. These pathogenic genes were involved in translational modification, signal transduction, and resistance to oxidative damage, which were relatively common virulence-associated factors in plant pathogenic fungi. Moreover, an abundance of gene clusters involved in secondary metabolism was found in *T*. *albobiverticillius*. The majority of these secondary metabolites were toxic polyketides, nonribosomal peptides, terpenes, and indoles that induce cell death in the host and contribute to disease development. The genomes contained many gene clusters, including T1PKS, NRPS-like, and NRPS. These secondary metabolites included alternariol, cichorine, ACT toxin II, and monascorubrin. This is consistent with previous reports that, in *T*. *marneffei*, the PKS gene *PM1* is responsible for the production of yellow mitorubrin pigments (mitorubrinic acid and mitorubrinol). As a result, we suggest that *T*. *albobiverticillius* has genomic characteristics that favor virulence and pigment production.

Genome sequencing establishes a crucial molecular basis and foundation for our subsequent investigations into the gene function of *T*. *albobiverticillius*. We have successfully concluded a comprehensive study on the influence of diverse medium components, temperature variations, and culture conditions on pigment synthesis. Within this process, we also conducted a transcriptome sequencing experiment. Leveraging the fully annotated, whole-genome data, we were able to conduct a comparative analysis with the reference transcriptome data, yielding remarkably comprehensive results. Furthermore, our strategy encompasses the exploration of genes associated with pigment synthesis. We intend to target specific genes, such as the PKS1 polyketide synthases, for knockout experiments and subsequent validation of gene function. While the validation of these specific genes is pending confirmation through first-generation sequencing outcomes, the comprehensive scope of whole-genome sequencing furnishes a robust underpinning and remarkable convenience for endeavors such as gene prediction, primer design, and gene cloning.

## 5. Conclusions

Here, we present the genome sequence, assembly, and annotation of *T*. *albobiverticillius*. The *T*. *albobiverticillius* genome contains a rich diversity of putative genes that are also found in other plant pathogens. Furthermore, this genome sequence holds the potential to facilitate the development of molecular markers for use in evolutionary research. As the first available *T*. *albobiverticillius* genome assembly, it is poised to enhance disease management and pigment production strategies.

## Figures and Tables

**Table 1:** Genome assembly statistics of *Talaromyces albobiverticillius* strain Tp-2.

Variables	Statistics
Genome assembly size (bp)	38,354,882
Number of contigs	14
Contigs N50 (bp)	4,594,200
Maximum contigs length (bp)	6,575,826
GC content (%)	45.78
BUSCO completeness (%) *	98.3
Fragmented BUSCOs (%)	0.3
Missing BUSCOs (%)	1.4
Total BUSCOs searched	758
SINEs (bp)	2322
LINEs (bp)	32,971
LTR (bp)	175,050
DNA transposons (bp)	116,497
Total length of Repeats (bp)	321,868
TR (bp)	244,896
Minisatellite DNA (bp)	161,135
Microsatellite DNA (bp)	18,152
Protein-coding genes	10,380
Mean gene length (bp)	1432

* BUSCO, Benchmarking universal single-copy orthologs; SINEs: Short interspersed nuclear elements; LINEs: Long interspersed nuclear elements; LTR: Long terminal repeat retrotransposons; TR: Tandem repeat.

**Table 2:** Genome assembly statistics of *Talaromyces albobiverticillius* and phylogenetically related *Talaromyces* taxa.

Genome Statistics	*Talaromyces* *albobiverticillius*	*Talaromyces* *atroroseus*	*Talaromyces* *islandicus*	*Talaromyces* *marneffei*	*Talaromyces* *verruculosus*
strain	Tp-2	IBT 11181	WF-38-12	ATCC 18224	TS63-9
host	*Punica granatum*	*Capsicum annuum*	*Triticum aestivum*	*Homo sapiens*	*Nicotiana tabacum*
country	China	Denmark	Germany	USA	China
sequencing technology	Oxford Nanopore	Illumina HiSeq	Illumina MiSeq	SOLiD	Illumina HiSeq
assembly size (Mb)	38.4	30.8	34.7	28.6	37.6
protein-coding	10,380	9523	9927	10,023	11,447
GC content (%)	46	46	46	46	46
unassigned genes ^1^	381	325	515	429	398
one-to-one orthologous ^2^	—	6396	5809	5871	6493

^1^ Unassigned genes were not assigned to any orthogroups of these five species genome data. ^2^ Orthologous genes compared to *T. albobiverticillius* genes.

**Table 3:** Gene functional annotation of *Talaromyces albobiverticillius* strain Tp-2.

Variables	Statistics
Protein-coding genes	10,380
Genes annotated by Pfam	7412
Genes annotated by KEGG	8844
Genes annotated by KOG	2160
Genes annotated by NR	9782
Genes annotated by Swiss-Prot	3657
Type I polyketide synthases genes	214
Cytochrome P450 genes	227
Virulence genes	400
Carbohydrate active enzymes	750
Transmembrane proteins	2057
Transport proteins	638
Secreted Protein	671

**Table 4:** Categories of carbohydrate-active enzymes in *Talaromyces albobiverticillius* strain Tp-2.

Categories	Families	Genes
Glycoside Hydrolases (GHs)	105	427
Glycosyltransferases (GTs)	37	121
Polysaccharide Lyases (PLs)	8	11
Carbohydrate Esterases (CEs)	10	40
Auxiliary Activities (AAs)	14	60
Carbohydrate-Binding Modules (CBMs)	12	91

**Table 5:** Putative genes of the Pathogen–Host Interactions database in *Talaromyces albobiverticillius* strain Tp-2.

Pathogen Species	PHI Database	Associated with Virulence ^1^
*Alternaria alternata*	10	8
*Aspergillus flavus*	15	15
*Aspergillus fumigatus*	147	83
*Beauveria bassiana*	45	30
*Bipolaris maydis*	11	11
*Botrytis cinerea*	41	18
*Candida albicans*	44	35
*Colletotrichum gloeosporioides*	10	8
*Colletotrichum graminicola*	7	6
*Cryptococcus neoformans*	40	29
*Fusarium graminearum*	366	118
*Magnaporthe oryzae*	163	117
*Sclerotinia sclerotiorum*	17	11
*Verticillium dahliae*	19	11
*Zymoseptoria tritici*	17	8

^1^ Genes associated with virulence, including loss, reduced, and increased virulence genes, chemistry targets, and effectors (plant virulence determinants).

## Data Availability

The read data of Oxford Nanopore and Illumina sequencing have been submitted to the NCBI Sequence Read Archive with accession number PRJNA835810. This Whole Genome Shotgun project has been deposited at DDBJ/ENA/GenBank under the accession JAMBUR000000000. The version described in this paper is version JAMBUR020000000.

## Supplementary Materials

The following supporting information can be downloaded at: https://www.mdpi.com/article/10.3390/jof9090909/s1, Figure S1: BUSCO assessment results of the *T. albobiverticillius* genome of the Tp-2 strain; Table S1: Gene ontology annotation of the *T. albobiverticillius* genome; Table S2: Carbohydrate-active enzyme annotation of *T. albobiverticillius* genome; Table S3: Transport protein annotation of *T. albobiverticillius* genome; Table S4: Secreted proteins annotation of *T. albobiverticillius* genome; Table S5: Homologous virulence genes of the *T. albobiverticillius* genome; Table S6: Putative genes in the PHI database of the *T. albobiverticillius* genome; Table S7: Secondary metabolites gene cluster annotation of the *T. albobiverticillius* genome.
