# Supplementary figures and images for: Genomic Sequence Resource of Talaromyces albobiverticillius, the Causative Pathogen of Pomegranate Pulp Rot Disease
Source: J Fungi (Basel). 2023 Sep 7;9(9):909. doi: 10.3390/jof9090909 (PMC10533087; doi:10.3390/jof9090909)

# BUSCO Assessment Results

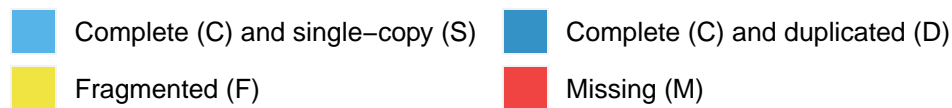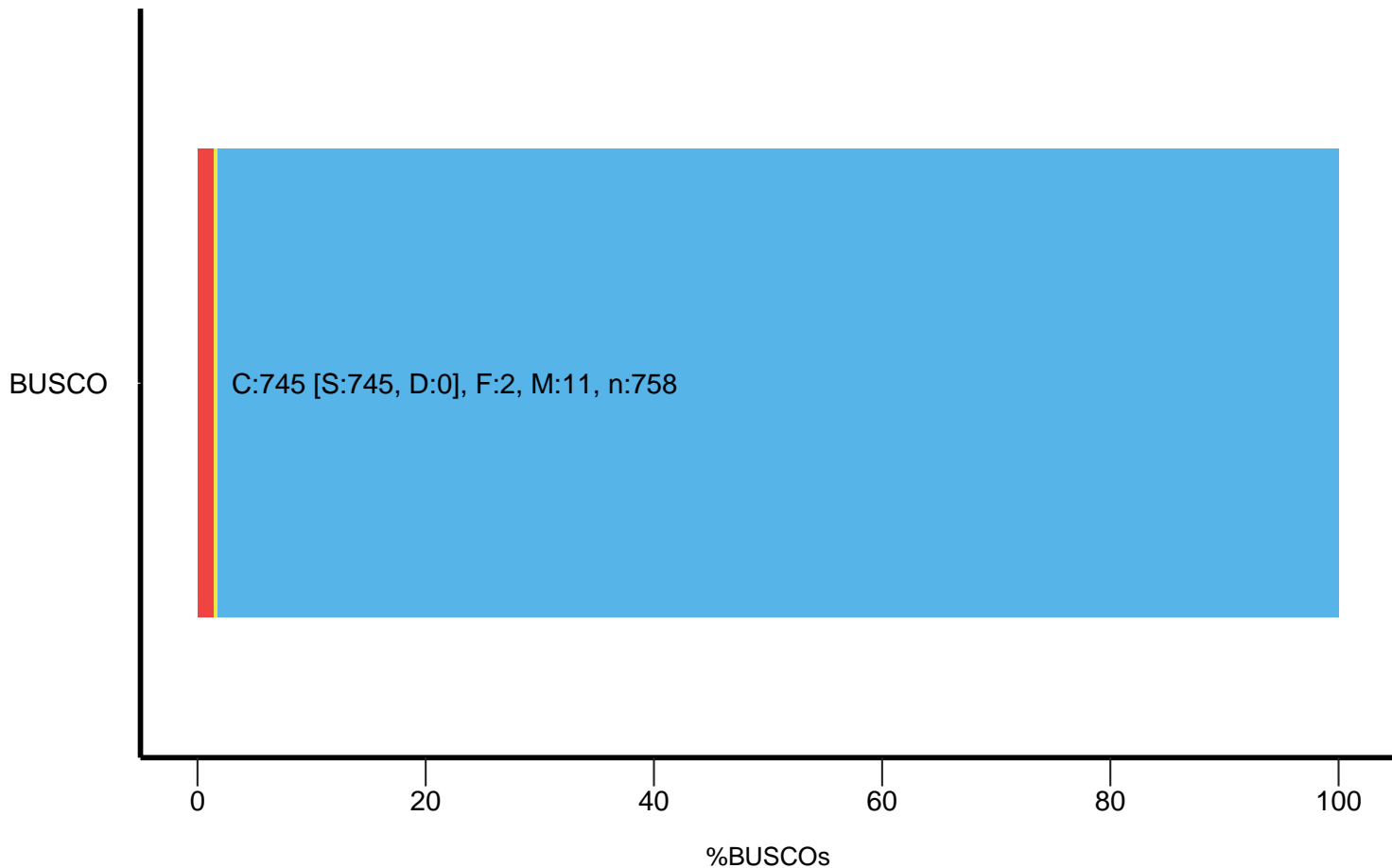

Supplement: Supplementary file 1 [file jof-09-00909-s001.zip › Supplementary/Figure S1.pdf]
